# Daily Life and Challenges Faced By Households With Permanent Childhood Developmental Disability in Rural Tanzania – A Qualitative Study

**DOI:** 10.1007/s10882-021-09809-6

**Published:** 2021-10-02

**Authors:** Joёlle Castellani, Omari Kimbute, Charles Makasi, Zakayo E. Mrango, Aggie T. G. Paulus, Silvia M. A. A. Evers, Pip Hardy, Tony Sumner, Augusta Keiya, Borislava Mihaylova, Mohammad Abul Faiz, Melba Gomes

**Affiliations:** 1 Department of Health Services Research, Care and Public Health Research Institute (CAPHRI), Maastricht University, Maastricht, The Netherlands; 2 Kilosa Station, National Institute for Medical Research, Kilosa, Tanzania; 3 Patient Voices Programme, Pilgrim Projects Limited, Landbeach, UK; 4 Dorcas Relief and Development, Handeni, Tanzania; 5 Health Economics Research Centre, Nuffield Department of Population Health, University of Oxford, Oxford, UK; 6 Institute of Population Health Sciences, Barts and the London School of Medicine and Dentistry, Queen Mary University of London, London, UK; 7 Mahidol Oxford Tropical Medicine Research Unit (MORU), Mahidol University, Bangkok, Thailand; 8 UNICEF/UNDP/World Bank/WHO Special Programme for Research & Training in Tropical Diseases (TDR), World Health Organization, Geneva, Switzerland

**Keywords:** Developmental disability, Poverty, Africa, Challenges

## Abstract

Severe developmental disability in children affects the life of the child and entire household. We conducted a qualitative study to understand how caregivers manage severe developmental disabilities in children in rural Africa. Families and six children (out of 15 children) who had serious permanent sequelae from a cerebral infection in Handeni, Tanzania, were contacted and invited to a workshop to recount their experience living with severe developmental disabilities. After consent, individual interviews were conducted first through recording of individual digital stories and then through individual semi-structured interviews. Pre-determined key categories were used to analyse the data. Our results showed that developmental disabilities required constant care and reduced the autonomy of the children. Schooling had not been attempted or was halted because of learning problems or inability to meet specialized school costs. Parents were under constant physical, emotional and financial stress. Their occupational earnings decreased. Some families sold their assets to survive. Others began to rely on relatives. Understanding the consequences of developmental disability helps to identify where social support should be focused and improved.

## Background

Severe developmental disability in children alters a child’s life and that of the family but the consequences have rarely been detailed. In developing countries, the impact might be greater because of poverty, difficult living conditions and poor access to care and rehabilitation. Consequently, children with severe developmental disability may have lower school attendance (Eide & Kamaleri, 2009; Filmer, 2008; Trani & VanLeit, 2010), lower educational attainment (Filmer, 2008; Mont & Cuong, 2011; Trani & Loeb, 2012) and fewer adult employment opportunities (Mitra, 2008; Mitra & Sambamoorthi, 2008; Trani & Loeb, 2012). Families with disability are generally poorer (Filmer, 2008), have fewer possessions (Mitra et al., 2011; Palmer et al., 2012), live in worse conditions (Eide & Kamaleri, 2009; Loeb & Eide, 2004) and experience a higher level of stress than families without disability (Mobarak, 2000). Disability is reported to tip households into poverty and transfer poverty to the next generation (Yeo & Moore, 2003).

Research on developmental disability has usually been quantitative, classifying the short and long-term clinical, neurological and cognitive impairments (Bangirana et al., 2016; Birbeck, 2013; Idro et al., 2016; Khandaker et al., 2014; Michaeli et al., 2014). The economic consequences for different types of disabilities have also been quantified for patients, households (Eide & Kamaleri, 2009; Filmer, 2008; Lamichhane & Kawakatsu, 2015; Trani & Loeb, 2012) and caregivers (Cheshire et al., 2010; Dambi et al., 2015; Sawyer et al., 2011). Challenges faced by caregivers and coping strategies to overcome these barriers have been described but there are quite limited data on the burden of caring for children with developmental disabilities in rural Africa, and how this burden changes as the child develops (Gona et al., 2011; Hartley et al., 2005; McNally & Mannan, 2013). We carrried out a qualitative study of adolescent children with serious permanent developmental sequelae ranging from major cognitive impairments to severe physical restrictions, or both to document the perceptions of caregivers on (1) their child’s development, (2) the treatment, (3) their emotional and physical burden, (4) their challenges and (5) their coping strategies over a 10-year period after the initial sequelae.

## Methods

### Study Participants

In 2014, the families of six children (out of 15 children) with persistent sequelae after a cerebral infection that had occurred at least 10 years earlier were asked for consent to meet, recount their stories and be interviewed about their lives in a 2-day workshop. All six parents/guardians consented and were reimbursed for accommodation, meals and transport costs.

The six children (four females and two males) were aged between 9 and 13 years (Table 1). Four children were accompanied by their mother, one by her aunt and one by his father. Two parents were married, three were separated or divorced and one was widowed. Five children had either convulsions or coma or both in the episode that caused developmental disabilities. Except for one child who had been hospitalized (child 4), none of the other children reported any hospital admissions since the initial episode, although they experienced uncomplicated malaria episodes that did not require hospital admission (child 1: 1 episode; child 3: 5 episodes; child 5: 4 episodes).

### Current Developmental Disabilities

All six children had one or more profound physical and/or cognitive disabilities (Table 2). Child 1 had limited cognition, was unable to speak, could not follow instructions and was reported to play only with much younger children. Child 2 could only walk on her knees and had elbow constrictions in her left arm. Child 3 had motor problems, abrupt changes of behaviour and partial hearing loss. Child 4 had epilepsy, dysconjugate gaze, impaired gait, made unsteady movements and frequently fell, could not articulate words, was doubly incontinent and was described as having limited cognition. Child 5 had problems with the right upper and lower limbs. Child 6 could not straighten his legs and therefore could not stand, had limited vision and hearing and was unable to speak; he was diagnosed with epilepsy and was described as having restricted cognition.

### Data Collection

The data were first collected through individual stories of the families, which were digitally recorded (Patient Voices, n.d.) and then through an individual semistructured interview. All data were collected during the 2-day workshop.

#### Digital individual stories

Each parent/guardian was free to choose specific moments of life regarding the illness/developmental disability of their child. They outlined what they wanted to say in their own words and were permitted to focus on their feelings, the impact of the illness/developmental disability and the challenges they faced associated with caring for a child with developmental disabilities (Patient Voices, 2014). The individual digital stories were recorded in Swahili by the parents/guardians and then translated and recorded in English by a local researcher fluent in both languages.

#### Semi-structured interviews

Once the digital stories were recorded, an individual semi-structured interview with parent–child pairs on the challenges of living with and caring for a child with developmental disabilities was completed. For this purpose, a questionnaire was used, containing open questions on seven topics on how developmental disability affects daily life: worries of the parents/guardians; the physical efforts of the carer; the effects of developmental disability on carer’s time; the child’s needs and medications used; social relationships; discipline/mood; and financial issues (Table 3). The interview questions and the seven topics were based on previous literature (Davis et al., 2010; Gona et al., 2011; Hartley et al., 2005; Murphy et al., 2007) and on the Behaviour Questionnaire for Parents – a questionnaire developed in East Africa adapted from the Kaufmann Assessment Battery for Children (Kaufman & Kaufman, 2004) which assesses behaviour difficulties in the home. Open questions (developed in English, translated into Swahili) on the seven topics, asked in the same sequence, guided the interview. The six interviews were conducted in Swahili by only one experienced bilingual researcher and each interview lasted about 30 minutes. Answers were immediately translated into English and transcribed, with clarifications on meaning sought, as necessary, during the workshop.

### Data Analysis

We analysed the data from both the digital stories and the semi-structured interview by using key categories that were pre-determined according to the seven topics of the semi-structured interview and previous research (Davis et al., 2010; Gona et al., 2011; Hartley et al., 2005; Murphy et al., 2007). Each digital story and interview transcription was read several times. Meaning units were extracted by the first author and copied into a spreadsheet where they were condensed and assigned a code. Each code was then placed under a pre-determined key category into a hierarchical frame.

## Results

The results are presented into five main themes, namely: (1) the child’s development, (2) the treatment, (3) the emotional and physical burden of the parents/guardians, (4) the challenges and (5) the coping strategies (Table 4). Each main themes were then separated into sub-topics.

### Child’s Development

Three main sub-topics emerged from the data: (1) autonomy, (2) communication and understanding and (3) behaviour and socialization.

#### Autonomy

The data showed that developmental disability affected the child’s autonomy. Most children with developmental disabilities were dependent on their parents/guardians who fed, dressed and bathed them.

For children with only physical disabilities who are aware of their disabilities, a lack of autonomy led to sadness, frustration, depression and anger especially when children were constantly dependent on somebody else for toileting and unable to do things that children of their age would normally do.

#### Communication and understanding

For children with limited mental capacity, understanding or retaining information was difficult. In some cases, communication was not possible when children were unable to speak and could not communicate that they were hungry, or needed to go to the toilet or that they were not feeling well. Constant supervision was often required because if left unattended, even for a few minutes, children might leave home and not know how to return.

#### Behaviour and socialization

The guardians described how developmental disabilities affected the behaviour and socialization of their child. Serious social problems were mentioned in relation to children with mental disabilities and epilepsy. Children with epilepsy or mental disabilities were socially disengaged. They exhibited unsociable behaviour and did not respond in ways which were normally expected; their responses and changes in facial expressions suggested that they did not know which behaviours were appropriate and which were not. This apparently led to social interaction problems with both adults and children.

For children with epilepsy or with frequent convulsions and emotional outbursts, mood changes were sudden and could greatly impact on the quality of life of the children and their families. Unpredictable outbursts were often difficult to control. Even for parents of children who are not mentally disabled, the attitude of their child might be unpredictable, extreme and uncontrollable which made it difficult to manage.

### Treatment

This topic was separated into two sub-topics: (1) epilepsy and (2) convulsions.

#### Epilepsy

Only one child who had epilepsy received regular treatment (child 4). Drugs for epilepsy are usually free and available from healthcare facilities. However some healthcare facilities, especially those that can manage epileptic cases, are located far away from communities, and therefore, access to treatment is difficult. Parents must walk long distances and may not have funds for transport.

#### Convulsions

The data showed that parents were not sure what to do when their child convulsed. Often parents did nothing or just put water on the child. However for one mother, traditional medicine was a solution. She was told by a traditional healer that the convulsions were caused because evil spirits were looking for her child, and therefore it was important to deal with them by changing the name of the child before healing could occur.

### Emotional and Physical Burden of the Parents/Guardians

This topic was separated into two sub-topics: (1) psychic pain and (2) physical health.

#### Psychic pain

All parents/guardians expressed fear, stress, depression, sadness and/or worry because of the developmental disability of their child. The normal stresses and worries of bringing up a child with developmental disability became even more important when the father had left the household and/or when the parents did not know if the condition of their child would improve.

With age and the hormonal and physical changes of adolescence, more difficulties were anticipated. Bringing up a female child was associated with increased level of stress, fear and worries as parents were concerned that their child would never be able to protect herself from her lack of understanding on sexual matters.

Fear of not being available or not knowing what to do when convulsions occurred was often expressed by carers. Convulsions caused the family to panic or be extremely sad, and parents/guardians said they feared the worst when the epileptic seizures ceased and the child fell into a deep sleep of unconsciousness.

#### Physical health

Parents/guardians described physical fatigue and back and/or chest pain caused by children who were unable to walk because this meant that they had to carry their child, usually on their back (children 2, 4 and 6). In addition, some mothers mentioned that they were often exhausted because they had to sleep in the same bed as their child which created discomfort.

### Challenges

This section had three main topics: (1) work and income, (2) poverty and (3) schooling.

#### Work and income

The child’s developmental disability influenced household’s life and limited income-generating activities of the family. The time required to take care of the child meant reduced time to work to earn an income and often the parents had to choose whether they should take care of their child or whether they should continue to work. The difficulties increased when the mother became the only sole economic provider because the father left the household.

#### Poverty

The data showed that because of a reduction in work, income decreased and households had a lower quality of life. The parents mentioned that they did not have the necessary funds to buy basic necessities such as food, medicine, school materials, furniture or clothes. In some cases, developmental disability pushed households into extreme poverty.

#### Schooling

Only two children (children 2 and 3) had ever gone to school; neither could continue (Table 1). Child 3, who had partial hearing loss, motor problems and mental disability, went to two different mainstream (normal) schools but as she was not able to cope, the schools did not wish her to continue. Because it is difficult for a child with developmental disabilities to go to a normal school, parents stated that they would like to enrol their child in a specialized school. However, education in such schools is fee based and unaffordable for families living in rural areas, especially when the mothers are divorced and the sole provider of the household. This was the case for child 2. She had started in a (specialized) boarding school, which did not require fees for the first year but was not allowed to stay beyond a year because her mother could not pay the tuition fees for the second year.

For the other four children – one with severe cognitive disability (child 1), two with epilepsy, limited cognition, physical disability and speech impairments (children 4 and 6) and one with severe lower limb weakness and restricted upper limb abilities (child 5) – school was never initiated (Table 1). For child 5, the walking distance to the school contributed to a decision not to enrol the child in school.

### Coping Strategies

Coping strategies was separated into two sub-topics: (1) dealing with work and (2) generating cash quickly.

#### Dealing with work

Most parents/guardians had to reduce their working time or had to stop working. In order to continue to work and generate an income, a solution mentioned by a widow mother (child 6) was to lock her epileptic child at home (who was moody and jumped around the house) while she was working at the farm. However, this strategy worked for some time when the child had fewer fits and was younger, but when the child started to convulse more often, he became a danger to himself when left alone. Another mother (child 2) who had to walk every day to her farm (1 hour each way) by carrying her child who could not walk on her back found a radical solution to continue working by moving home to her farm even if this meant a decrease in her quality of life. For other families, trying to keep the same level of work (and income) as before was made possible through the help of siblings although this implied the siblings dropped out of school.

#### Generating cash quickly

The data revealed that in order to survive, some parents/guardians decided to sell their personal assets. For others, the extended family was an important source of finance.

## Discussion

Three major consequences of developmental disability emerged from the data: significant emotional and physical burden of caring for children with developmental disabilities, a decrease in work and unrelenting financial pressure on family income, assets and savings, and no or reduced school attendance for the child.

Caring for a child with developmental disabilities placed an emotional and physical burden on families. Children were dependent, needed assistance with daily tasks and activities and had little prospect of later improvement. Children with epilepsy had unpredictable seizures. Parents felt the constant pressure of being physically present for their dependent child. Consequently, as confirmed by other studies, all parents were exhausted, stressed, depressed and worried (Cheshire et al., 2010; Dambi et al., 2015; Davis et al., 2010; Sawyer et al., 2011). Some expressed desperation. Difficulties in communicating and understanding the needs of children with mental disability increased parental stress, anxiety, and depression both about the current circumstances (Hartley et al., 2005) as well as about the future. As mentioned elsewhere, physical disability increased the physical burden of carers looking after a child (Brehaut et al., 2004; Dambi et al., 2015; Davis et al., 2010; Hartley et al., 2005). Stress and distress were voiced by mothers whose spouse had abandoned the household, leaving them to support the whole family. Long-term developmental disability appeared to damage carers’ health, destroy family structures, and reduce the quality of family life (Mobarak et al., 2000).

Developmental disability has financial costs. Parents either left their occupations or worked less because their child required full-time care. As a result of a reduction in occupational work, household income decreased leading to financial problems in purchasing necessities such as food or clothes (Gona et al., 2011; Hartley et al., 2005; McNally & Mannan, 2013). To cope with extra expenses, the families reported selling their assets thus pushing households who were already poor into extreme poverty (Mitra & Sambamoorthi, 2008; Yeo & Moore, 2003). Exiting from this poverty trap remains difficult as families may never have the necessary resources to do so.

Most children in our study never went to school. Children with epilepsy had difficulties in attending normal schools especially when the epileptic seizures were frequent, unexpected, or were followed by coma, drowsiness, confusion, irritability, aggression, dizziness, loss of concentration, memory problems and mood swings. Seizures caused memory loss and confusion, and children appeared to have significant problems with learning, language or behaviour throughout their childhood, in addition to social stigma (bullying, anxiety, depression, self-consciousness). For both children with physical and/or cognitive disability, the distance to the school, and the lack of infrastructure, adequate learning materials and trained teachers, made access to normal schools even more difficult (ACPF, 2014). In Uganda, the negative attitude of normal schools contributed to lack of enrolment (Hartley et al., 2005). Specialized schools are usually fee-based and often inaccessible both physically and financially. Similar results were found in other studies in Tanzania and Uganda (Hartley et al., 2005; McNally & Mannan, 2013). Lack of education led to parental concerns about the future of their child (Hartley et al., 2005). Lower school participation led to lower educational attainment which translated into lower employment opportunities and lower income in adulthood (Filmer, 2008; Yeo & Moore, 2003).

To improve the quality of life of both children and households and reduce stress, the risk of depression, worries, and isolation, social support has been shown to be effective (Ainbinder et al., 1998; Bray et al., 2017; Kerr & McIntosh, 2000). However, the households only received family support. Social support can be found at specialized centres, such as community-based rehabilitation centres (Cameron et al., 2005; CCBRT, n.d.), even in Tanzania. Free home-based therapies for children with physical and/or mental disabilities as well as regular home and school follow-up visits are offered (Cameron et al., 2005; CCBRT, n.d.). Children are taught how to increase their independence (e.g. washing, moving from their bed to a chair) and how to help the household (e.g. washing the dishes) (Cameron et al., 2005). However, community-based centres are rare and most centres are located in major (urban) cities which are not accessible or expensive for households living in rural areas. Another option to support children with developmental disabilities and their families is the involvement of community rehabilitation facilitators to provide home visits, exercises and training in activities of daily living (ADL—i.e. eating; bathing; getting dressed; toileting; transferring and continence) (Chappell & Johannsmeier, 2009). This kind of intervention can provide ADL independence and increase social integration, self-esteem, self-confidence, mobility and acceptance of developmental disability (Chappell & Johannsmeier, 2009).

Our study is rare in examining how caregivers manage severe childhood developmental disabilities that occurred early in infancy and continued into adolescence without resolution. The ability to probe deeply through qualitative research on how developmental disability affects the family is a strength. Thematic saturation was achieved with the small number of children. However, the small number of children and parents interviewed does not provide a complete picture of the challenges in living with developmental disability. More topics might have emerged with a larger sample of parent–child pairs. A further limitation is that only one male guardian participated in the study and male views on the consequences of developmental disability, especially on stress, work and financial burdens, may differ from the views of female guardians. The qualitative interviews were not tape-recorded because ethical clearance was received only for recording digital stories (Patient Voices, 2014). Finally, only children with severely developmental disabilities took part in our research, and therefore, this study does not represent the challenges of caring for children with milder developmental disabilities.

## Conclusions

Severe developmental disability affects not only the life of the child but that of the whole household. It increases physical, emotional and financial burdens of the child and carers. Children have very limited educational support—either from normal or specialized schools—and access to rehabilitative home-based support is inaccessible or unaffordable. Parental income is reduced because time for caring is increased and consequently households that are poor get pushed further into poverty.

## Figures and Tables

**Table 1:** Characteristics of the children and accompanying guardians

	Child 1	Child 2	Child 3	Child 4	Child 5	Child 6
Respondent	Mother	Mother	Mother	Aunt^a^	Father	Mother^b^
Marital status of respondent	Separated/divorced	Separated/divorced	Separated/divorced	Married	Married	Widowed
Sex of the child	Female	Female	Female	Female	Male	Male
Age of the child in months at index episode^c^	23	24	33	32	17	20
Age of the child in years during the interview (April 2014)	11	9	11	11	9	13
CNS (convulsions, coma/altered consciousness) at index episode^c^ (based on observation)	Convulsions only	Convulsions only	Convulsions + coma/altered consciousness	Convulsions + coma/altered consciousness	Convulsions + coma/altered consciousness	No CNS
Malaria status at index episode^c^	Malaria	Malaria	No malaria	No malaria	Malaria	Unknown
Developmental disability at discharge during the index episode^c^	Cerebellar damage	Right sided hemiparesis	Right sided hemiparesis	Severe epilepsy, abnormal speech, decreased hearing, and ataxia	Right sided hemiparesis and decreased hearing	Left sided hemiparesis with later severe epilepsy
History of illness and hospital admissions between index episode and follow-up	No history of hospitalization post index episode. However the child has a history of 1 febrile episode since the index episode (no CNS symptoms) and recovered fully	No reported history of episodes requiring hospital admission post index episode	No hospital admissions post index episode. However, the child has a history of 5 febrile episodes, 2 of malaria since the index episode. Recovered fully after treatment	One hospital admission post index episode. The child was unable to sit/stand/walk, and was identified as having severe anaemia	No hospital admissions post index episode. However the child has a history of 4 febrile episodes since the index episode and was treated for malaria. The child recovered	No hospital admissions post index episode and no history of febrile episodes since the index episode
Schooling of the child	None	Halted schooling. Was admitted at a special school but the mother could not pay fees	Halted schooling. The school refused continuation because the child could not follow the teaching	None	None	None
Number of siblings	4	5	2	10	7	6

*CNS* central nervous system

a The mother of the child could not participate in the workshop. Therefore, she asked her sister to participate

b The aunt of the child also participated with the mother in the semi-structured interview

c Original episode

**Table 2:** Current developmental disabilities of the children

Child 1	Child 2	Child 3	Child 4^a^	Child 5	Child 6^b^
- Speech disorder - Limited mental capacity - Decreased hearing - Has mood swings - Plays with very young children	- Severe elbow constriction of right arm which does not reach level of left - Severe constriction of lower limbs - Walks on her knees	- Partial hearing loss - Cerebellar dysfunction on finger-nose test - Fine finger movements with difficulty or failure - Has abrupt changes of behaviour or sudden inappropriate changes from usual behaviour - Mother indicates significant improvement as can now use right upper limb and walks normally	- Dysconjugate gaze - Does not walk normally (flat footed, with hands open, at times stomping gait) - Falls frequently - Limited mental capacity - Abrupt changes in behaviour - Double incontinence - Shows no emotions - Unsteady movements - Groans	- Tends to fall frequently due to wasting of right lower limb - Right lower limb looks shorter than left - Right upper limb not swinging, fixed to body while walking - Failure to achieve fine finger movements with right hand - Father indicates improvement as the child can now walk and pick up objects with his right hand	- Cannot speak - Vision and hearing abnormal - Physically handicapped - Cannot stand or straighten his legs - Spastic left upper limbs - Mentally handicapped/severe cognitive disability - Sits staring - Double incontinence

a Vision and hearing could not be tested as the child couldn’t/wouldn’t cooperate

b Fine finger exercises, heel-toe walking, walking neurological exams could not be done

**Table 3:** Examples of questions of the case report form

Categories	Questions
Worries of the parents/guardians	Could you please tell me more about how the developmental disability of your child affects you? Examples of elements to be included: More stress, sadness and worries? How do you see the future for you, your child and your family?
Physical efforts of the carer	Could you please tell me how you are physically affected? (For instance, how do you deal if your child cannot walk?) Examples of elements to be included: More physical efforts? If yes, do you have any health problems due to these additional physical efforts? Are you feeling exhausted by these additional efforts?
Effects of developmental disability on carer’s time	Could you please tell me more about how your time is affected by the developmental disability of your child when comparing to a child who has no developmental disability? Examples of elements to be included: When you are at home, are you always together with him/her the whole day? How do you manage your time at home? Is some of your time allocated to basic stuff such as dressing, feeding, toileting, sleeping etc.? In which areas? Do you have some extra help? If yes, what kind of “other” help do you have?
Child’s needs and medications used	Could you please tell me how you deal with your child’s needs, medication and special treatment? Examples of elements to be included: How do you understand your child’s needs (because cannot speak)? Need of special treatment? What about the toilet? What help does your child need? Need to take drugs regularly?
Social relationships	Could you please let me know how your relations with your family and other people have been affected and how your child is socially affected? Examples of elements to be included: How has your child been with his/her siblings over the past month? Do they argue much? Are there other children around here who your child can play with? If yes, is (s)he willing to play with them? Are they playing together? Have you or your child already been socially excluded? Have you already encountered some difficult situations within the family (e.g. father left)?
Discipline/mood	Could you please tell me how you deal with discipline? Do you think your child is more difficult than a child with no developmental disability? Is (s)he often angry, does (s)he scream a lot? Examples of elements to be included: Mood changes frequently? Has explosive, angry outbursts? What does your child do when angry? Does (s)/he scream and kick? How many times in the past month?
Financial issues	What are the financial consequences for your household? Examples of elements to be included: Did you need to give up or change your job? Or did you need to work less? If you have any other children, how are they affected? Do they also need to allocate their time to take care of the child? Maybe they cannot go to school or do their homework? Did some of your children need to give up school in order to work? Have you already encountered a lack of food?

**Table 4:** Themes and sub-themes of the results

Themes	Subtheme	Examples of codes	Supporting data
Child’s development	Autonomy	- Dependence - Frustration	*‘..She [the child] cannot talk. She cannot walk properly. She is doubly incontinent. She cannot ask for food. She moans a lot. She is completely dependent. ’* (Aunt of child 4) *‘My child complains that she cannot walk and fetch water like other children… She is also very depressed because she cannot go to the toilet by herself. She needs my help and that makes her angry. ’* (Mother of child 2)
	Communication and understanding	- Understanding - Communication - Unable to speak - Supervision	*‘If you ask her [the child] to bring a cooking pot, she brings back a broom. ’* (Mother of child 1) *‘My niece cannot… say when she needs to go to the toilet. Therefore, when she needs to go to the toilet, she will stay where she is. ’* (Aunt of child 4)
	Behavior and socialization	- Unsociable - Mood change - Does not laugh - Does not smile - Outburst - Angry - Scream - Cry - Social interactions	*‘She [the child] likes to be alone. She is never playing with other children or with her siblings, and she does not like to be touched by people she does not know… She does not laugh and does not smile. Most of the time, she stands or sits and makes some noise. If her siblings are coming to play with her, she will hit them because she does not want to be with them. If some children want to play with her, she will go somewhere else. If they follow her, she will hit them as well. ’* (Aunt of child 4) *‘When she [the child] is angry, she cries, goes behind the house and undresses herself. ’* (Mother of child 1) *‘When in a bad mood, she [the child] does notallow any guests to come in the house… She is in a bad mood on average once a day. ’* (Mother of child 3) *‘Since my son was 4 years old, he has sometimes an explosive, angry outburst… For instance, he will throw and break cups. ’* (Father of child 5)
Treatment	Epilepsy	- Free drugs - Access to healthcare facilities - No improvement	*‘We [the household] receive the drugs [for epilepsy] from the district hospital. The drugs are free but we have to pick them up at the hospital once a month. It takes about 3 hours to walk there. And the drugs are even not useful because my niece is not improving. ’* (Aunt of child 4)
	Convulsions	- Confusion/Not sure what to do - Traditional healer	*‘The mother then puts water on her [the child] and fans her. ’* (Aunt of child 4 – once convulsions are over) *‘ When my child has convulsions, I do nothing. I don’t give anything. I just watch and wait.’* (Mother of child 6) *‘…The convulsions continued after we returned home [from the hospital]. We sought assistance from the traditional healer who advised that we change the child’s name. Before she was known by one name, and now she is known by a different name.’* (Mother of child 1)
Emotional and physical burden of the parents/guardians	Psychic pain	- Stress - Worries - Sadness - Depression - Fear - Scare	*‘I feel very depressed. The [developmental] disability of my child made me sad… lam also anxious because I don’t know if she [the child] will be able to talk in the future.’* (Mother of child 1) *‘l am worried… because when she [the child] will get her periods, she will not understand… and not know what to do. I am also scared that someone could abuse her and get her pregnant.’* (Mother of child 1) *‘ Very often my niece collapses and has convulsions. Then nothing happens. It is silent and we [the family] think that she is going to die.’* (Aunt of child 4)
	Physical health	- Chest and/or back pain - Fatigue	*‘In the past, I was walking every day to the farm, carrying my child on my back (1 hour for one way)… and now I have some pain somewhere close to my back.’* (Mother of child 2) *‘She [the child] sleeps in the same bed as her mother. She often wakes up in the middle of the night, and sits and makes some noise.’* (Aunt of child 4)
Challenges	Work and income	- Less time for work - Reduced income	*‘ I was selling food at a gold mine but I had to stop because I wanted to be close to my child.’* (Mother of child 3) *‘Before [developmental disability] I was farming and selling food in a small business. When my child became disabled, I had to stop selling food. l am now only farming…. ‘* (Mother of child 2)
	Poverty	- No money for necessities - Extreme poverty - Single - parent families	*‘There is no bed. We [the mother and the child] just sleep on the floor… I am just using medicine (when I am sick) because I cannot take my son and myself to hospital… They ask for money.’* (Mother of child 6) *‘I have some food problems… I even cannot afford to buy pens or exercise books [for school] for my two sons. ‘* (Mother of child 2) *‘We cannot buy any clothes anymore’* (Aunt of child 4)
	Schooling	- No integration - Coping problems - Unaffordable - Distance	*‘You see, I cannot make the requirements of the school… I was expected to provide 30,000 TSh (~12 USD), 3 measures of cereals, 20 kilos of beans and a uniform.’* (Mother of child 2)
Coping strategies	Dealing with work	- Locking the child at home - Moving closer to work - Child work	*‘… I decided to move there [to the farm] because it was too exhausting to carry her [the child] every day.’* (Mother of child 2) *‘…[my] other [child] finished primary school. (S)he doesn’t go to school anymore because (s)he has to farm.’* (Mother of child 1)
	Generating cash quickly	- Selling furniture and necess ities - Selling the house - Family support	*‘After I stopped my work at the gold mine, my finances went down. I had to sell everything in my house.’* (Mother of child 3) *‘Once, my 3 children were sick at the same time. Thus, I had to sell my small house in the village so that I could afford the treatment.’* (Mother of child 2) *‘Sometimes, we [the family] receive financial assistance from relatives. These relatives … give us money so that we can get a motorcycle when we have to go somewhere.*’ (Aunt of child 4)

*TSh* Tanzanian shillings, *USD* US dollars

## Data Availability

The digital stories are available under the following link: https://www.patientvoices.org.uk/savingbrains.htm.
